# The Design and Experimentation of a Corn Moisture Detection Device Based on Double Capacitors

**DOI:** 10.3390/s24051408

**Published:** 2024-02-22

**Authors:** Changjie Han, Yurong Wang, Zhai Shi, Yang Xu, Shilong Qiu, Hanping Mao

**Affiliations:** College of Mechanical and Electrical Engineering, Xinjiang Agricultural University, Urumqi 830052, China; aster691832507@163.com (Y.W.); shizhai-001@126.com (Z.S.); xuyang_928@126.com (Y.X.); maohp@ujs.edu.cn (H.M.)

**Keywords:** online detection, corn moisture, double capacitors, simulation optimization, temperature compensation, porosity

## Abstract

Detecting the moisture content of grain accurately and rapidly has important significance for harvesting, transport, storage, processing, and precision agriculture. There are some problems with the slow detection speeds, unstable detection, and low detection accuracy of moisture contents in corn harvesters. In that case, an online moisture detection device was designed, which is based on double capacitors. A new method of capacitance complementation and integration was proposed to eliminate the limitation of single data. The device is composed of a sampling mechanism and a double-capacitor sensor consisting of a flatbed capacitor and a cylindrical capacitor. The optimum structure size of the capacitor plates was determined by simulation optimization. In addition to this, the detection system with software and hardware was developed to estimate the moisture content. Indoor dynamic measurement tests were carried out to analyze the influence of temperature and porosity. Based on the influencing factors and capacitance, a model was established to estimate the moisture content. Finally, the support vector machine (SVM) regressions between the capacitance and moisture content were built up so that the R2 values were more than 0.91. In the stability test, the standard deviation of the stability test was 1.09%, and the maximum relative error of the measurement accuracy test was 1.22%. In the dynamic verification test, the maximum error of the measurement was 4.62%, less than 5%. It provides a measurement method for the accurate, rapid, and stable detection of the moisture content of corn and other grains.

## 1. Introduction

Maize is one of the main field crops around the world. It has great production potential and high economic benefits. It has been used for feed and various industrial purposes [1], which leads to an important strategic position in ensuring food security. However, excessive moisture in corn can lead to mildew, food safety accidents, and economic losses, which is not conducive to transportation and storage [2,3]. Moreover, for grain harvesting machinery, moisture content measurement not only provides a reference for the working parameters of threshing and cleaning components; it also provides a basis for precise yield measurement. It is also the development direction of intelligent agricultural equipment in the future.

There are two methods, direct and indirect, for measuring the moisture content [4,5]. The direct method can be divided into the drying method and chemical method, but it has the disadvantage that it is time-consuming and inefficient [6]. Thus, it is not appropriate for online detection. The indirect method involves converting the received data from grains into the moisture content, including the resistance method, near-infrared spectroscopy, the dielectric property method [7], etc. The advantages of these methods are that they are quick and portable by using non-destructive testing, and they are suitable for online detection [8].

Previously, microwaves were applied to grain moisture measurement, but was easily influenced by the surrounding environment [9]. R. Thakur developed a prototype multi-grain moisture meter based on the capacitive sensing technique, and its measurement accuracy was found to be 1% [10].

Mathew G. developed a high-accuracy frequency-domain sensing probe for use in obtaining dielectric measurements of materials suitable for work ranging from 300 MHz to 1 GHz [11]. Huaiyu Liu developed an online detection device for the moisture content based on the capacitance method, and this device has good accuracy and stability [12]. Based on the dielectric properties, K. B. Kim developed an online moisture meter prototype based on RF impedance and moisture density [13]. Zhenran Gao designed a parallel resonance circuit of a voltage-controlled oscillator and high-frequency inductance–capacitance (LC) for signal frequency division and conditioning [14]. M.E. Casada tested a new fringing field capacitive (FFC) sensor to determine its suitability and accuracy for moisture content measurements in grain. The measured sensor accuracy is close to that of laboratory instruments [15]. G. C. Zoerb developed a device to estimate the moisture content of grain by measuring the resonant frequency of a capacitor consisting of two metal plates [16]. Bahram Besharati developed a cylindrical capacitor with an oscillator circuit to measure the moisture content of five seeds; its accuracy is 97% [17]. Regardless, simple data conversion cannot meet the required detection accuracy. Kok Yeow You developed the empirical polynomial models to predict the gravimetric moisture content of rice based on measured reflection coefficients using a vector network analyzer [18]. Xueqiang Liu designed a structural modular neural network by combining the BP neurons and the RBF neurons at the hidden layer to predict the moisture content in the grain drying process [19]. In a word, these methods only use one sensor to measure the moisture of grain, which will cause bigger errors in complex environments.

The current study aimed to develop the moisture content detection of corn harvesters and even combine harvesters. In other words, an online detection method was developed based on double capacitors that has a simple structure, high measurement accuracy, fast detection speed, and low cost. A detection system was built with the STM32 chip microcomputer. We explore the influence of porosity and temperature on capacitance by selecting three kinds of corn kernel varieties with different sizes as the test objects. A relationship model between plate capacitance, cylinder capacitance, temperature, porosity, and moisture content was constructed. It can improve the detection accuracy of moisture content [20]. After that, design experiments were conducted to verify the model. Finally, the design of an online detection device for moisture content was completed with higher stability, accuracy, and speed and lower cost. Moreover, it provides a reference method for grain online detection.

## 2. Design of the Corn Moisture Sensor

This section will describe the detection principle and how to design this sensor to detect moisture. It aims to develop an online moisture content detection device to realize online moisture detection.

### 2.1. Detection Principle

The main components of a capacitor are metal plates and an intermediate medium. When the dielectric constant of the medium changes [21], the capacitance will change. The dielectric constant of water is almost 80, far higher than corn [22]. As a result, the relative dielectric constant is different at different levels of moisture. Dielectric change causes capacitance change. In this case, the online device usually measures capacitance to estimate the moisture of grains. By measuring the capacitance, the moisture of corn will be predicted.

**Figure 1:**
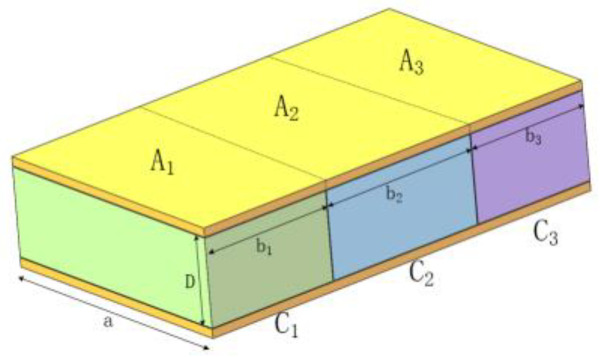
Equivalent model of flatbed plate medium.

However, many factors will influence the capacitance measuring. Taking the flatbed capacitor as an example, the medium can be divided into corn, water in the corn, and air [23]. The equivalent model diagram of this plate medium is showed in Figure 1. The relative total area of the two parallel plates is the sum of the relative areas of the three parts. Thus, the capacitance of every part can be calculated by Equations (1) and (2).

(1) $$A=ab_{1}+ab_{2}+ab_{3}=A_{1}+A_{2}+A_{3}$$

(2) $$C=C_{1}+C_{2}+C_{3}=\frac{\varepsilon_{0}A}{D}(\frac{A_{1}}{A}\varepsilon_{1}+\frac{A_{2}}{A}\varepsilon_{2}+\frac{A_{3}}{A}\varepsilon_{3})$$

A—relative total area (m^2^);

A_1_—relative area of corn (m^2^);

A_2_—relative area of water (m^2^);

A_3_—relative area of air (m^2^);

a—total length (m);

C—equivalent total capacitance (F);

C_1_—equivalent capacitance of corn (F);

C_2_—equivalent capacitance of water (F);

C_3_—equivalent capacitance of air (F);

ε_0_—dielectric constant in vacuum (ε_0_ = 8.85 pF/m);

ε_1_—dielectric constant in corn (F/m);

ε_2_—dielectric constant in water (F/m);

ε_3_—dielectric constant in air (F/m);

D—plate spacing (m).

As we all know, no grain contains no water. So, we take A_1_ and A_2_ as a whole. In this way, the relative total capacitance can be calculated by Equations (3)–(6).

(3) $$\left\{ \begin{array}{l} M_{1}=M(1-W)=V_{1}\rho_{1}\\ M_{2}=MW=V_{2}\rho_{2}=V_{2} \end{array}\right.$$

(4) $$A_{1}+A_{2}=\frac{M_{1}/\rho_{1}+M_{2}/\rho_{2}}{D}=\frac{M_{1}+M_{2}\rho_{1}}{\rho_{1}D}$$

(5) $$C=\frac{\varepsilon_{0}A}{D}(\frac{M-MW+\rho_{1}MW}{\rho_{1}AD}\varepsilon +\frac{A_{3}}{A}\varepsilon_{3})$$

(6) $$C=k_{0}\frac{\varepsilon (\rho_{1}-1)M}{\rho_{1}AD}W+k_{0}\frac{M}{\rho_{1}AD}+k_{0}e\varepsilon_{3}$$

M—total mass of corn (Kg);

M_1_—mass of dry corn (Kg);

M_2_—mass of water (Kg);

W—moisture content of corn (%);

V_1_—volumes of dry grain (m^3^);

V_2_—volumes of water (m^3^);

ρ_1_—density of dry grain (Kg/m^3^);

ρ_2_—density of water (ρ_2_ = 1 Kg/m^3^);

ε—dielectric constant of grain.

In Equation (5), $A_{3}/A$ is the ratio of the plate volume between the grain and the entire volume of the material containing the pores, also known as porosity, and $\varepsilon_{0}A/D$ is the structural constant. Assuming that $e=A_{3}/A$, then $k_{0}=\varepsilon_{0}A/D$. Thus, Equation (5) can be rewritten as Equation (6).

According to Equation (6), it can be seen that the capacitance can be converted into the moisture content by using the flatbed capacitor. The moisture will be calculated once the capacitance measurement is finished. However, changes in temperature and porosity will affect the dielectric constant [24]. In that case, the capacitance will change, which will cause deviation in the moisture estimation. Thus, both temperature and porosity are necessary for accurate estimation. The temperature sensor is required to compensate for the measurement deviation caused by temperature. Given the shape of corn, it is divided into three types—large, medium, and small—to minimize the influence of porosity. Grains are divided according to porosity. A value greater than or equal to 0.42 is considered as a large grain; a value greater than or equal to 0.37 but less than 0.42 is considered as a medium grain; and a value between 0.33 and 0.37 is considered as a small grain.

### 2.2. Design of the Double Capacitors

#### 2.2.1. Design of Double Capacitors: Selection

Since the capacitance measurement has a simple structure, strong adaptability, and fast speed, it was chosen as the sensing element. There are three typical capacitors: flatbed capacitors, cylindrical capacitors, and single-plane capacitors. The first capacitor can store more charges, but it is greatly affected by the edge electric field. For the second type, the edge effect is small, but the capacitance is small, which can easily cause measurement errors. The capacitance value of the third is small and susceptible to external interference, which is not suitable for complex scenes in the field.

**Figure 2:**
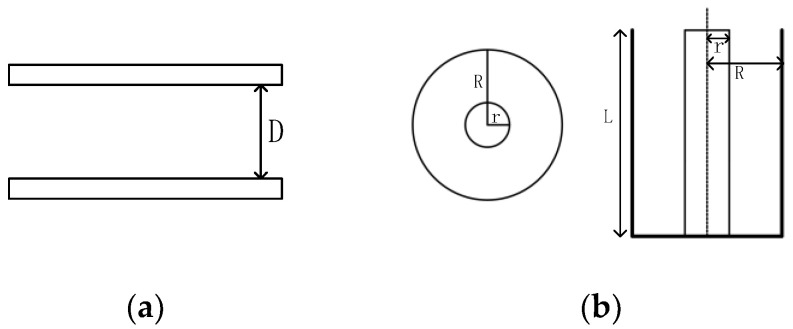
Two capacitor structures. (**a**) Flatbed capacitor; (**b**) cylindrical capacitor.

Based on the advantages and disadvantages of the three capacitor types, a combination of flatbed plates and cylindrical structures was selected to design a capacitive moisture detection sensor. It eliminates the limitations of a single datum. The sketches of the two plate structures are shown in Figure 2, Where D is the spacing between the plates, R is the outer radius of the cylinder, and r is the inner radius.

#### 2.2.2. Design of the Capacitor: Optimization

For the double capacitors, the capacitance is affected by the edge effect of the plate easily, which leads to the nonlinearity of the output [25]. For reducing the edge effect, the analysis software COMSOL Multiphysics 6.0 (COMSOL) was used to model the two capacitors and the air domain in three dimensions [26]. The plate material is copper, and the electrostatic field was used as the physical field. Finally, the simulated capacitance value of the capacitor is solved by the solver.

The edge effect is related to the constructional dimension of the capacitor. The constructional dimension will be changed by the control variable method. The simulation value is obtained according to the theoretical calculation formula. The degree of the edge effect will be reflected by the ratio C/C_0_ (hereinafter referred to as the capacitance ratio) of the simulation value and the theoretical value.

**Figure 3:**
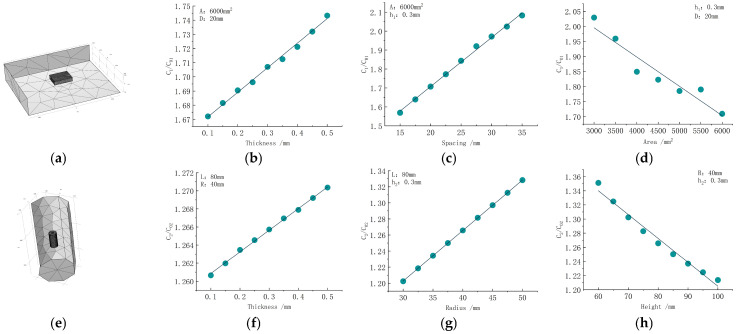
The relationship between plate structure parameters and edge effect: (**a**) flatbed meshing; (**b**) relationship between thickness and edge effect; (**c**) relationship between spacing and edge effect; (**d**) relationship between area and edge effect; (**e**) cylinder meshing; (**f**) relationship between thickness and edge effect; (**g**) relationship between outer radius and edge effect; (**h**) relationship between height and edge effect.

In the theoretical formula, for the flatbed capacitor, the plate area, distance, and thickness are the main factors affecting the edge effect. Similarly, the outer radius (the ratio of the inner and outer radius is a fixed value), height, and thickness are the factors of the cylindrical capacitor. The grid division of the two capacitors and the relationship between each factor and the edge effect are shown in Figure 3.

It can be seen from Figure 3 that, for the flatbed capacitor plate, the thickness and distance are positively correlated with the edge effect, and the area is negatively correlated. With the increase in thickness and distance, the electric field distribution at the edge of the two plates is not uniform, which increases the influence of the edge effect. When the plate area increases, the influence of the edge effect decreases. For the cylindrical capacitor, the relationship between the thickness and outer radius and the edge effect is positively correlated, and the relationship between the height and the edge effect is negatively correlated. When the thickness and outer radius are increased, the edge effect increases. When the height increases, the edge effect decreases.

The single-factor experiment shows that the constructional dimension is related to the edge effect. In order to obtain the optimal parameters to reduce the nonlinear influence caused by the edge effect, the constructional dimension is optimized and the optimal parameters are found by response surface analysis in the Design-Expert 13 software. The thickness (h_1_), distance (D), and area (A) of the flatbed capacitor were selected as experimental factors. The thickness (h_2_), outer radius (R), and height (L) of the cylinder capacitor were selected as the same. The capacitance ratio is used as the experimental index. The Box–Behnken (BBD) experimental design method was used to carry out the orthogonal test simulation. The level of experimental factors is shown in Table 1, and the analysis of variance is reported in Table 2.

**Figure 4:**
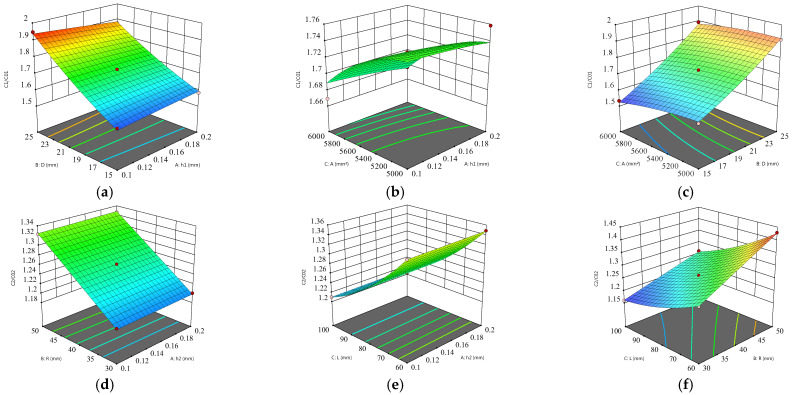
Response surface of interaction of various factors. (**a**) The influence of spacing and thickness on capacitance ratio. (**b**) The influence of area and thickness on capacitance ratio. (**c**) The influence of area and spacing on capacitance ratio. (**d**) Effect of thickness and radius on capacitance ratio. (**e**) Effect of thickness and height on capacitance ratio. (**f**) Influence of radius and height on capacitance ratio.

It can be seen from Table 2 that for the parallel plates, the influence of the distance (D) on the edge effect is extremely significant, and the area (A) is significant. For the cylindrical plates, the influence of the outer radius (R) and height (L) is extremely significant. The response surface [27] (as shown in Figure 4) of each factor and the capacitance ratio of the plate are obtained by analyzing the simulation test data.

According to the single-factor experiment and response surface, an optimization model can be derived. Among them, the thickness is selected to be the smallest, and other parameters select the optimal value within the selected range to minimize the edge effect. Considering the processing, the optimal value is adjusted to an integer. The optimal parameters obtained by using the software are as follows: The optimal values of the plate are h_1_ = 0.2 mm, D = 20 mm, and A = 6000 mm^2^. The optimal values of the cylinder are h_2_ = 0.1 mm, R = 40 mm, and L = 100 mm.

### 2.3. Design of the Software and Hardware Circuits

#### 2.3.1. Design of the Hardware Circuits

**Figure 5:**
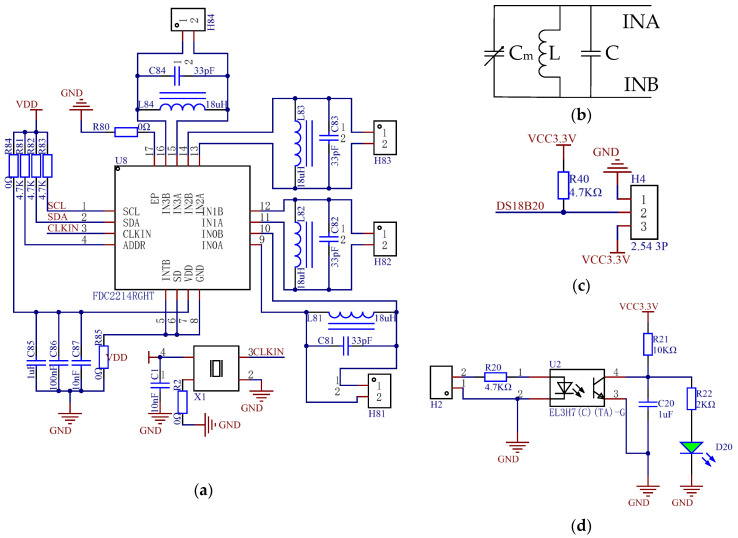
Main detection module hardware schematic circuits. (**a**) Capacitor detection module circuit. (**b**) Capacitance measurement schematic circuit. (**c**) Temperature measurement circuit. (**d**) Full state detection circuit.

The hardware circuits (as shown in Figure 5) mainly include a capacitance measurement circuit, a temperature measurement circuit, a full material state circuit, and other circuits such as the motor drive circuit, as well as data transmission.

(7) $$f_{m}=\frac{1}{2\pi \sqrt{L(C+C_{m})}}$$

(8) $$C_{m}=\frac{1}{L{\left(2\pi f_{m}\right)}^{2}}-C$$

L—fixed inductance (H);

C—fixed capacitance (F);

C_m_—capacitance value to be measured (F);

f_m_—frequency of the capacitance to be measured (Hz).

According to the calculation formula, the capacitances of both capacitors are calculated using the Keil uVision5 software. Finally, the moisture content of the corn is calculated by calibration.

The temperature detection circuit (as shown in Figure 5c) consists of a pull-up resistor and a temperature signal. A temperature sensor was used to detect the temperature in the sampling chamber and transmit the temperature to the microcomputer. The full material state switch (as shown in Figure 5d) was used to judge whether the sampling chamber was filled with materials. The optocoupler isolation converts the 24 V signal into a low-level signal that the microcomputer can receive and determines whether the material is filled by lighting the diode. The detection method has good insulation and an anti-interference ability.

#### 2.3.2. Design of the Software

**Figure 6:**
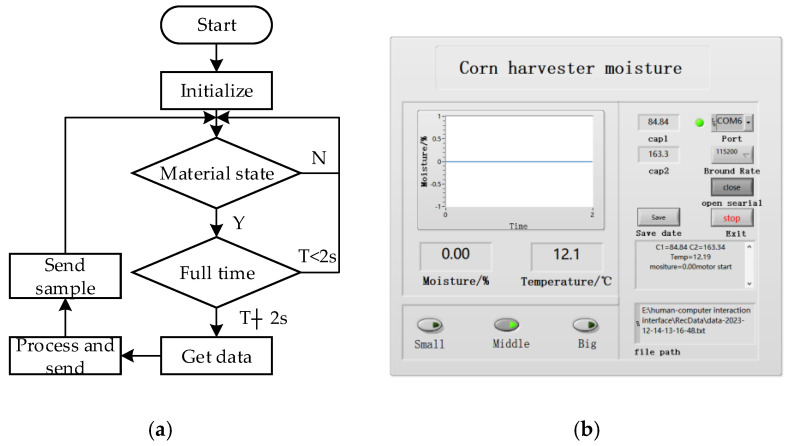
Design of the software. (**a**) Detection system program flow chart. (**b**) Upper computer interface.

The acquired data are transmitted to the upper computer through the serial interface for display and storage. The detection system (as shown in Figure 6a) mainly includes three stages: full material detection, data acquisition and processing, and sample return. STM32F103C8T6 was used as the microcomputer. Keil uVision5 was the development environment and the programming language was C. Finally, the data are uploaded to the upper computer through the serial port.

The upper computer uses the labview2017 software. The main functions (as shown in Figure 6b) include the display and storage of data such as moisture and temperature, the selection of grain size, and the trend of moisture.

### 2.4. Overall Design of Moisture Detection Device

**Figure 7:**
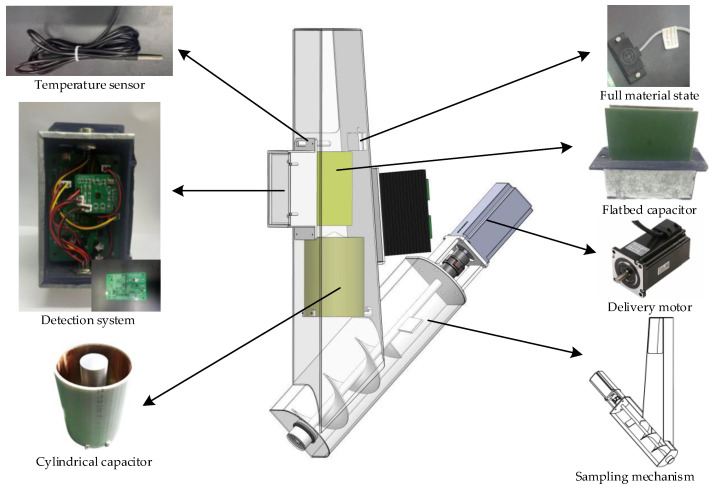
Overall design of moisture detection device.

Aimed at the problems of poor stability and low accuracy in detecting moisture, an online detection device (as shown in Figure 7) based on double capacitors was designed. This device is mainly composed of a temperature sensor, a moisture content detection system, a cylindrical, a full material state switch, a flatbed capacitor, a delivery motor, and a sampling mechanism. It is installed on the side of the harvester lift. The moisture content detection system uses a flatbed capacitor and a cylindrical capacitor to obtain capacitances. It also uses a temperature sensor to obtain the temperature and a full material state switch to judge the material state in the chamber.

The sampling mechanism is mainly divided into a sampling part and a delivery part. The sampling part is composed of a feed inlet and a chamber, where the material falls into. It accumulates until it fills the whole chamber. The upper narrow and lower wide chamber can prevent the material from suspending. The delivery part is composed of a sample delivery chamber, a sample delivery winch, and a delivery motor. The motor is a step motor, the torque is 3.6 N·m, and the power supply voltage is 24 V. When the material inside the elevator passes through the sampling port, part of it falls into the sampling chamber. When the chamber is filled, the detection system begins to work. After completion, the microcomputer drives the delivery motor to send the material back to the elevator. This mechanism realizes the intermittent sampling and continuous dynamic detection process of a corn grain harvester under operating conditions.

**Figure 8:**
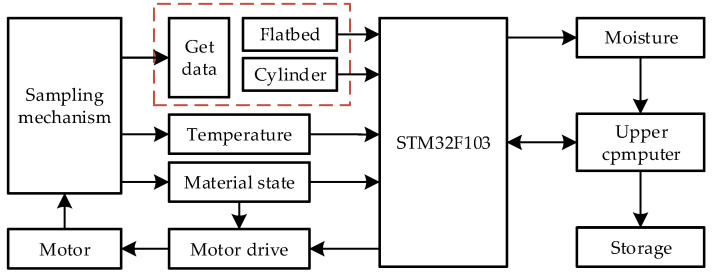
Working schematic diagram of online moisture content detection device.

The working principle of this online detection device is illustrated in Figure 8. When the material fills the sampling chamber, the full material state sensor is triggered and timing begins. When time exceeds two seconds, it is considered that the chamber has been filled. At this moment, the capacitance acquisition module begins to measure the capacitance, and the temperature sensor obtains the current temperature; the current moisture content is calculated by processing unit. Finally, these data will be displayed and stored in the upper computer. After that, the motor starts to send the material back to the inside of the lifting conveyor. The rapid measurement of corn moisture content with high stability and precision is realized through intermittent sampling and dynamic detection.

## 3. Materials, Analysis, Sensor Calibration, and Performance Tests

### 3.1. Materials, Single-Factor Analysis, and Sensor Calibration

#### 3.1.1. Materials

The change in the capacitance reflects the change in the moisture of corn. Moreover, this change is affected by the temperature and porosity. Therefore, three kinds of grain varieties with different grain sizes were selected, and the two factors were determined. The test data were collected and analyzed to achieve the purpose of calibration.

The relationship between porosity, temperature, double capacitance, and moisture was obtained by experiments. Three kinds of grain varieties with different sizes were selected. We calculated the porosity of corn using the measuring cup method. We randomly selected a certain amount of corn kernels and put them into a measuring cup until they covered the entire cup. Then, we added water to the cup. The volume of water divided by the total volume is the porosity of corn. The measured porosity of large grains is 0.42, that of medium grains is 0.37, and that of small grains is 0.33. The corn kernels were poured into the sampling port and accumulated. After the test was completed, the grains were discharged through the discharge port by the motor, and the capacitance and temperature data were recorded. The real water content was obtained by the rapid drying method [28], and the relationship between each factor and water content was obtained using the model. The test material corn kernel was selected from the corn of the Changji Hui Autonomous Prefecture Military Farm; the corn grain samples were not screened, and the impurities and broken grains were retained to simulate the real harvesting state of the harvesting machinery.

**Figure 9:**
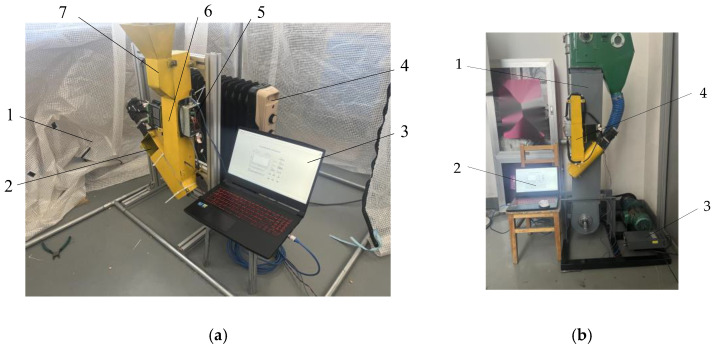
Laboratory calibration test. (**a**) Offline test: 1—greenhouse; 2—discharge port; 3—upper computer; 4—heater; 5—detection system; 6—sampling and feeding mechanism; 7—feeding port; (**b**) online test: 1—elevator; 2—upper computer; 3—power; 4—corn moisture detection device.

The change in porosity will affect the capacitance. In this study, the same moisture and capacitance are different at different levels of porosity, resulting in deviation in the results. Thus, in view of the influence of porosity on capacitance, a test of the influence of porosity on capacitance was designed. Three kinds of sizes were selected to prepare moisture contents in the range of 17–32% by adding water to achieve different moisture contents, and the effect of porosity on capacitance was tested in a greenhouse (as shown in Figure 9a) at about 30 °C. Each group of data was measured 50 times, and the average value was taken as the measured value and recorded. The real moisture was obtained by the rapid drying method. The test data were analyzed in origin2022.

In addition to the porosity factor, the dielectric constant of the material is easily affected by the temperature when this method is used to measure the moisture of the material, and it in turn affects the moisture detection. In order to explore the influence of temperature on capacitance, a test was designed. Small corn with a water content of 17.45% was selected, and the capacitance values at different temperatures were obtained by greenhouse heating. The capacitance at each temperature was measured continuously 50 times, and the average value was taken as the capacitance value at this temperature. The final fitting results were verified by an online test (as shown in Figure 9b).

#### 3.1.2. Factors Analysis

**Figure 10:**
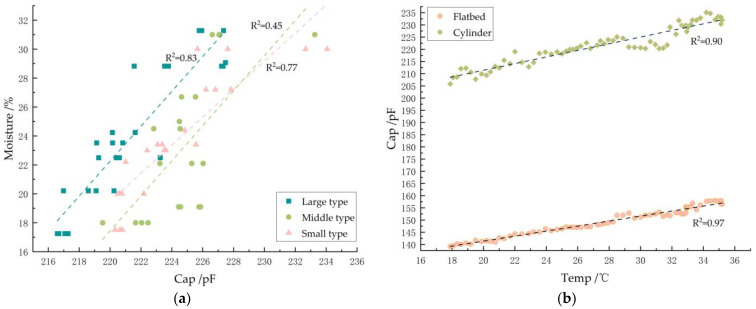
Single-factor analysis. (**a**) The relationship between cylinder capacitance and moisture under different porosity levels. (**b**) Effect of temperature on capacitance.

There are three kinds of relationships (as shown in Figure 10a) under porosity. It can be seen that, with the increase in the water content, the capacitance also shows an increasing trend, but the water content and capacitance show a discrete relationship. The lowest linear goodness of fit is 0.45, so it does not have a single linear relationship. The main reason is that the accumulation state of each grain in the sampling room is different, which is manifested as the change in porosity, resulting in different dielectric constants. Therefore, there is a large error in the detection of moisture by a single capacitor. As shown in Figure 10b, it can be seen that the capacitance increases linearly with the increase in temperature at the same porosity and the same water content. Therefore, temperature is an important factor affecting the accurate measurement of water content.

#### 3.1.3. Sensor Calibration

Through the analysis, it can be seen that the influence of the temperature factor is linear, but that of the porosity is not. Therefore, this study uses MATLAB R2016a to analyze the data and curve fitting [29]. Taking the temperature and two capacitances as the input variables and the water content as the output variable, support vector machine regression is used to fit the relationship between the temperature and capacitance and the water content at three porosity levels. The fitting equations are as follows:

Large type:

(9) $$y=-101.13+0.37x_{1}+0.46x_{2}-0.98x_{3}$$

Medium type:

(10) $$y=-14.76-0.16x_{1}+0.33x_{2}-0.29x_{3}$$

Small type:

(11) $$y=-16.18+0.07x_{1}+0.19x_{2}-0.37x_{3}$$

x_1_—flatbed capacitance (pF);

x_2_—cylinder capacitance (pF);

x_3_—temperature (°C).

Among them, the R^2^ of the large type is 0.96, that of the medium type is 0.95, and that of the small type is 0.91. By writing the fitting formula of the moisture of different grain sizes into the single-chip microcomputer program, and bringing in the values of each parameter, the moisture content of corn grains is solved at this time.

### 3.2. Performance Tests

#### 3.2.1. Stability Test

In order to verify the stability of this detection system, the corn was placed in the moisture content detection device, and the small type with a moisture content of 30.46% was selected. The moisture of the corn was repeatedly measured five times using the online moisture content detection system and is recorded in Table 3.

It can be seen that the standard deviation of the measurement results is 1.09% after five repetitions, and the moisture content detection device can achieve stable detection.

#### 3.2.2. Accuracy Test

In order to verify whether the accuracy of the system meets the necessary requirements, corn samples with different moisture contents were randomly prepared, and the samples were put into the detection device to measure their moisture content. Then, the samples were dried to obtain the true value of the moisture content of the samples. The measured moisture content and actual moisture content of the samples were recorded. Each group was measured five times. The average value was taken as the test result, which was compared with the drying method. The large-grain corn samples were selected to carry out the corn grain circulation flow detection test. The test results are shown in Table 4.

The precision measurement test shows that the maximum relative error is 1.22%, less than 5%, which meets the detection requirements, so this device can be used for moisture content detection.

#### 3.2.3. Dynamic Verification Test

In order to verify the practicability and reliability of the online detection device, the online dynamic precision measurement test of corn grain was carried out in the hangar of Xinjiang Agricultural University on 3 October 2023.

The online detection device of corn moisture content in this study was installed on the outside of the lifter and entered the detection device through the grain feed inlet. During the experiment, the moisture content measurement results were recorded every time, and the samples were taken out to dry the moisture content. Then, the recorded values were compared with the measured values. Every test was repeated five times, and the online dynamic precision measurement test results are shown in Table 5.

The online test showed that the maximum relative error of the measurement result was 4.62%, which is less than the general measurement requirement of 5%. Among them, due to the vibration caused by the movement of the hoist, a certain error was caused. So, the moisture content detection can be realized.

## 4. Conclusions

(1) The influence of porosity and temperature on the capacitance value was analyzed. At the same porosity, the capacitance is linear with temperature. At different porosities, the capacitance value is different and the linear relationship between the single capacitance and moisture content is poor. Therefore, the capacitance and temperature at different porosities are fitted with the moisture content. The fitting results are all greater than 92%, and the measurement error is within 5%, which meets the requirements of grain moisture content detection.
(2) Aimed at the problems of the poor detection stability and low accuracy of the current grain moisture content detection device in harvesters, a dual-capacitor detection device was designed. The complementarity and integration between the two groups of capacitors were used to improve the detection stability and accuracy. The structure size of the plate was optimized using simulation software to reduce the nonlinear effect caused by the edge effect of the capacitor.

## Figures and Tables

**Table 1:** Test factor level table.

Class	Factors	−1	0	1
Flatbed capacitor	h_1_/mm	0.10	0.15	0.20
	D/mm	15	20	25
	A/mm^2^	5000	5500	6000
Cylinder capacitor	h_2_/mm	0.1	0.15	0.2
	R/mm	30	40	50
	L/mm	60	80	100

h_1_ and h_2_ are plate thickness, mm; D is the plate spacing, mm; A is the relative area of the plate, mm^2^; R is the outer radius of the cylinder, mm; L is the height of the cylinder, mm.

**Table 2:** Analysis of variance table.

Factors	Source of Variance	Quadratic Sum	Degree of Freedom	Mean Square Deviation	F-Value	*p*-Value
Flatbed capacitor	Model	0.2350	9	0.0261	49.35	0.0042
	A-h_1_	0.0002	1	0.0002	0.4308	0.5584
	B-D	0.2231	1	0.2231	421.73	0.0003
	C-A	0.0072	1	0.0072	13.64	0.0344
	AB	0.0018	1	0.0018	3.42	0.1614
	AC	9.000E−06	1	9.000E−06	0.0170	0.9045
	BC	0.0005	1	0.0005	0.9358	0.4047
	A^2^	8.036E−06	1	8.036E−06	0.0152	0.9097
	B^2^	0.0008	1	0.0008	1.55	0.3013
	C^2^	0.0004	1	0.0004	0.7162	0.4596
	residual error	0.0016	3	0.0005	49.35	
	total variation	0.2366	12			
Cylinder capacitor	Model	0.0719	9	0.0080	849.43	<0.0001
	A-h_2_	0.0000	1	0.0000	1.67	0.2872
	B-R	0.0334	1	0.0334	3545.73	<0.0001
	C-L	0.0367	1	0.0367	3897.22	<0.0001
	AB	0.0000	1	0.0000	0.0000	1.0000
	AC	6.400E−07	1	6.400E−07	0.0680	0.8111
	BC	0.0010	1	0.0010	108.16	0.0019
	A^2^	0.0000	1	0.0000	0.0000	1.0000
	B^2^	3.657E−07	1	3.657E−07	0.0389	0.8563
	C^2^	0.0006	1	0.0006	67.35	0.0038
	residual error	0.0000	3	9.408E−06		
	total variation	0.0720	12			

*p* < 0.01 is extremely significant; *p* < 0.05 is considered significant; *p* > 0.1 is not significant.

**Table 3:** Stability test.

Group	Moisture/%	Average	Standard Deviation
1	30.1	30.16	1.09
2	31.1		
3	29.9		
4	30.0		
5	29.7		

**Table 4:** Precision measurement test.

Group	Measured Value/%	Real Value/%	Related Error/%
1	22.8	22.6	0.88
2	24.2	24.5	−1.22
3	26.7	27.0	−1.11
4	28.5	28.2	1.06
5	31.2	31.5	−0.95

**Table 5:** Online dynamic precision measurement test.

Class	Group	Measured Value/%	Real Value/%	Related Error/%
Small	1	23.1	23.3	−0.86
	2	24.2	23.3	3.86
	3	24.1	23.3	3.43
	4	23.8	23.3	2.15
	5	22.9	23.3	−1.72
Medium	1	25.4	24.4	4.10
	2	25.0	24.4	2.46
	3	25.5	24.5	4.08
	4	25.1	24.5	2.45
	5	25.8	25.0	3.20
Large	1	24.8	24.1	2.90
	2	24.9	24.3	2.47
	3	24.9	24.1	3.32
	4	24.9	23.8	4.62
	5	24.9	25.7	−3.11

## Data Availability

The dataset used in this research is available upon valid request to any of the authors of this research article.
